# True and False Mesmerism

**Published:** 1845-10

**Authors:** 


*True and False Mesmerism.*—Dr. Charles Radclyffe Hall, of London, in a series of Articles in the Lancet, on the Rise, Progress, and Mysteries of Mesmerism, in all ages and countries, deduces the following conclusions:

"Of the alleged results of mesmeric processes, I believe there are

*Proved*—Quietude; composure; sleep.

*Probable, but requiring confirmation*—Traction; muscular

---

rigidity; convulsions; heightened sensibility; diminished sensibility; double consciousness.

*Possible, but not very probable*—Insensibility to severe pain, for a given length of time, at pleasure.

*Impossible, as far as anything can be so*—Clairvoyance; intuition; prevision; community of thought; involuntary and complete subjection of mind to the mesmeriser.

And, lastly, I believe that we have not a shadow of evidence in support of the existence of any new agency, whether designated mesmeric, magnetic, occult, or by any other name."

Up to the present hour, we know of no Medical Journal, either in Europe or in this country, that looks upon mesmerism with the least allowance.—*Southern Medical and Surgical Journal.*

